# TaqMan real-time polymerase chain reaction for detection of *Ophidiomyces ophiodiicola*, the fungus associated with snake fungal disease

**DOI:** 10.1186/s12917-015-0407-8

**Published:** 2015-04-15

**Authors:** Elizabeth Bohuski, Jeffrey M Lorch, Kathryn M Griffin, David S Blehert

**Affiliations:** United States Geological Survey, National Wildlife Health Center, Madison, WI USA; Department of Pathobiological Sciences, School of Veterinary Medicine, University of Wisconsin-Madison, Madison, WI USA

**Keywords:** *Chrysosporium* anamorph of *Nannizziopsis vriesii* (CANV), Emerging disease, *Ophidiomyces ophiodiicola*, Real-time PCR, Snake fungal disease

## Abstract

**Background:**

Fungal skin infections associated with *Ophidiomyces ophiodiicola*, a member of the *Chrysosporium* anamorph of *Nannizziopsis vriesii* (CANV) complex, have been linked to an increasing number of cases of snake fungal disease (SFD) in captive snakes around the world and in wild snake populations in eastern North America. The emergence of SFD in both captive and wild situations has led to an increased need for tools to better diagnose and study the disease.

**Results:**

We developed two TaqMan real-time polymerase chain reaction (PCR) assays to rapidly detect *O. ophiodiicola* in clinical samples. One assay targets the internal transcribed spacer region (ITS) of the fungal genome while the other targets the more variable intergenic spacer region (IGS). The PCR assays were qualified using skin samples collected from 50 snakes for which *O. ophiodiicola* had been previously detected by culture, 20 snakes with gross skin lesions suggestive of SFD but which were culture-negative for *O. ophiodiicola*, and 16 snakes with no clinical signs of infection. Both assays performed equivalently and proved to be more sensitive than traditional culture methods, detecting *O. ophiodiicola* in 98% of the culture-positive samples and in 40% of the culture-negative snakes that had clinical signs of SFD. In addition, the assays did not cross-react with a panel of 28 fungal species that are closely related to *O. ophiodiicola* or that commonly occur on the skin of snakes. The assays did, however, indicate that some asymptomatic snakes (~6%) may harbor low levels of the fungus, and that PCR should be paired with histology when a definitive diagnosis is required.

**Conclusions:**

These assays represent the first published methods to detect *O. ophiodiicola* by real-time PCR. The ITS assay has great utility for assisting with SFD diagnoses whereas the IGS assay offers a valuable tool for research-based applications.

## Background

Skin infections are among the most common diseases observed in captive snakes [1,2]. While fungi are often implicated in many of these infections through histopathologic analyses, identifying the exact causative agent can be difficult. Traditionally, culture analyses were the standard for determining the likely causes of dermatomycoses, but such analyses are complicated by the abundance and diversity of fungi naturally found on the skin of reptiles [2,3]. In addition, relatively little work has been conducted to identify which of these fungi are medically important [2]. The veterinary literature contains numerous case reports implicating particular fungi as the causes of dermatitis in snakes, but few offer compelling evidence that the fungi grown in culture truly represent the agent observed in supporting histopathology [2]. In many cases, the etiology of skin infections in snakes has likely been incorrectly attributed to common environmental microorganisms and such misdiagnoses may lead to unsuccessful treatment regimens and serve to confound our understanding of the causes of such infections in captive snakes.

Fungi formerly grouped within the *Chrysosporium* anamorph of *Nannizziopsis vriesii* (CANV) complex are among the few species that have been repeatedly associated with dermatological disease in reptiles (summarized by [2,4]), and at least one species has been shown through infection trials to act as a primary pathogen in healthy chameleons [5]. Recent phylogenetic analyses of the CANV complex have revealed several new taxa [6,7]. One of these species, *Ophidiomyces* (formerly *Chrysosporium*) *ophiodiicola*, was described for the first time in 2009 after its isolation from a captive snake with a facial granuloma [8]. However, upon re-examining isolates formerly classified as CANV, Sigler et al. [6] found that *O. ophiodiicola* has been associated with emerging skin infections in captive snakes for the last several decades. Many additional cases of dermatitis associated with *O. ophiodiicola* are thought to have been incorrectly attributed to bacteria or other fungi [2], and *O. ophiodiicola* may be one of the most common, albeit overlooked, causes of skin infections in captive snakes.

Since 2006, *O. ophiodiicola* has also been isolated from wild snakes with severe, and often fatal, infections in the eastern U.S. [9,10]. These emerging infections, referred to as snake fungal disease (SFD), are currently considered a serious threat to some snake populations [9,11]. However, recent attempts to study the prevalence, distribution, and impacts of SFD on wild snakes have been hampered by the lack of rapid, cost-effective, and reliable laboratory tests to detect *O. ophiodiicola*.

Confirming presence of *O. ophiodiicola* in association with skin lesions can be problematic due to the difficulty of isolating the fungus in culture. Isolation is particularly challenging when small samples such as biopsies or scale clippings are collected, and euthanasia of the animal is frequently required to obtain larger samples sufficient for confirming the presence of *O. ophiodiicola*. Even when the fungus is isolated in culture, identification of *O. ophiodiicola* based on morphological characteristics is often unreliable. Furthermore, the fungus is relatively slow-growing such that the process of successful isolation and identification can take weeks and may be dependent upon laboratory expertise. Given the recent emergence of SFD in wild snakes and the clinical importance of fungal dermatitis in captive snakes, a rapid and more sensitive tool for the detection of *O. ophiodiicola* is needed.

Here we describe the development and qualification of two TaqMan real-time polymerase chain reaction (PCR) assays that reliably detect *O. ophiodiicola* from small pieces of skin tissue collected from live snakes. The PCR tests target either the multi-copy internal transcribed spacer region (ITS) or intergenic spacer region (IGS) of *O. ophiodiicola* and are extremely sensitive, highly specific, and yield results in less than 24 hours. These assays offer utility in assisting with diagnosis of SFD in both wild and captive snakes in addition to providing an important research tool for better understanding the biology of the fungus and ecology of this disease.

## Methods

### DNA extraction, amplification, and sequencing

All fungal cultures were grown at 24°C on Sabouraud dextrose agar containing chloramphenicol and gentamicin or dermatophyte test medium. After 7 to 21 days (depending on the growth rate of a particular isolate), approximately 5 to 10 mg of mycelia were scraped off of the medium and placed into 600 μl lyticase solution [1 M sorbitol, 100 mM EDTA, 200 U lyticase (Sigma-Aldrich, St. Louis, MO), and 14 mM beta-mercaptoethanol], ground with a pestle, and incubated at 30°C and 500 rpm for 1 hour. Fungal protoplasts were pelleted by centrifugation at 500 x g for 10 min, the supernatant removed, and genomic DNA (gDNA) extracted using the Gentra®Puregene® Tissue Kit (Qiagen Inc., Valencia, CA) according to the “solid tissues protocol” and omitting proteinase K and RNase treatments.

The gDNA extracted from isolates of *O. ophiodiicola* used in this study (see Table 1) was quantified at the University of Wisconsin Biotechnology Center using an Invitrogen™ Qubit™ Fluorometer (Life Technologies, Carlsbad, CA). To ensure that the gDNA extracted from all other fungal isolates (*i.e.*, non-*O. ophiodiicola* isolates listed in Tables 1 and 2) was of sufficient quantity and quality for downstream PCR applications, the internal transcribed spacer region (ITS) was amplified using the fungal-specific primers ITS1-F and ITS4 [12] under the following cycling conditions: 94°C for 3 min; 40 cycles of 94°C for 1 min, 53°C for 1 min, and 72°C for 3 min; and a final extension at 72°C for 10 min. GoTaq® Flexi DNA polymerase (Promega Corporation, Madison, WI) was used for the PCR reactions following the manufacturer’s instructions, and 5 μl template (diluted 1:10) was added per 25 μl reaction. All such reactions yielded strong bands of the correct size, indicating that fungal gDNA had been successfully extracted.

**Table 1:** **Fungal isolates for which ribosomal RNA regions were analyzed to develop** ***Ophidiomyces ophiodiicola*** **-specific PCR assays**

			GenBank Accession No.*	
**Isolate**	**Species**	**ITS Reference**	**ITS**	**IGS**
CBS 122913	*Ophidiomyces ophiodiicola*	[8]	EU715819	KP691505
UAMH 6218	*Ophidiomyces ophiodiicola*	[6]	KF477227	KP691506
UAMH 6642	*Ophidiomyces ophiodiicola*	[6]	KC884267	KP691507
UAMH 6688	*Ophidiomyces ophiodiicola*	[6]	KF477228	KP691508
UAMH 9985	*Ophidiomyces ophiodiicola*	[6]	KF477230	KP691509
UAMH 10296	*Ophidiomyces ophiodiicola*	[6]	KF477232	KP691510
UAMH 10768	*Ophidiomyces ophiodiicola*	[6]	KF477234	KP691511
UAMH 10769	*Ophidiomyces ophiodiicola*	[6]	KF477235	KP691512
UAMH 10949	*Ophidiomyces ophiodiicola*	[6]	KF477236	KP691513
UAMH 11295	*Ophidiomyces ophiodiicola*	[6]	KF477237	KP691514
UAMH 10212	*Chrysosporium indicum*	this study	KP691483	KP691515
CBS 629.79	*Chrysosporium* sp.	this study	KP691484	KP691516
UAMH 10352	*Nannizziopsis guarroi*	[6]	KF477208	KP691517
UAMH 10417	*Nannizziopsis infrequens*	[6,22]	AY744467	KP691518
UAMH 3527	*Nannizziopsis vriesii*	[6]	KF477198	KP691519
UAMH 10439	*Paranannizziopsis australasiensis*	[6]	KF477218	KP691520
UAMH 10693	*Paranannizziopsis californiensis*	[6]	KF477224	KP691521
UAMH 8392	*Pseudoamauroascus australiensis*	[23]	AJ131787	KP691522

CBS = isolates obtained from the Centraalbureau voor Schimmelcultures.

UAMH = isolates obtained from the University of Alberta Microfungus Collection and Herbarium.

ITS = internal transcribed spacer region of the ribosomal RNA gene complex.

IGS = intergenic spacer region (3′ and 5′ ends) of the ribosomal RNA gene complex.

*All IGS DNA sequences were newly generated for this study.

**Table 2:** **Fungal isolates from snakes that were used to assess specificity of** ***Ophidiomyces ophiodiicola*** **PCR assays**

**Isolate**	Species*	**ITS GenBank Accession No.**
44736-30-02-02	*Alternaria* sp.	KP691485
24411-01-01-02	*Arthroderma/Trichophyton* sp.	KP691486
44736-44-01-01	*Arthroderma/Trichophyton* sp.	KP691487
24833-01-01-01A	Ascomycete sp.	KP691488
24821-01-02-01	*Aspergillus* sp.	KP691489
44736-02-01-01	*Beauveria* sp.	KP691490
24392-01-03-04	*Bionectria* sp.	KP691491
24266-04-05-01	*Cladosporium* sp.	KP691492
24392-01-02-02	Hypocreales sp.	KP691493
24415-01-02-02	*Isaria* sp.	KP691494
24266-01-03-02	*Metarhizium* sp.	KP691495
24266-01-01-01A	*Mucor* sp.	KP691496
44736-32-02-01	*Myriodontium* sp.	KP691497
44736-01-01-01	*Paecilomyces* sp.	KP691498
44736-43-05-04A	*Penicillium* sp.	KP691499
24281-01-01-01	*Penicillium* sp.	KP691500
44736-32-01-01	*Pseudogymnoascus* sp.	KP691501
44736-03-02-01	*Purpureocillium lilacinum*	KP691502
24281-01-02-01	Trichocomaceae sp.	KP691503
24826-01-01-01A	*Trichoderma* sp.	KP691504

*Identifications were based on DNA sequencing of the internal transcribed spacer region (ITS).

To generate sequence data for the IGS of *O. ophiodiicola* isolates, primers CNL12 [13] and CNS1 [14] were used to amplify the approximately 2,800 bp region under the following cycling conditions: initial denaturation at 98°C for 2 min; 47 cycles of 98°C for 10 sec, 50.5°C for 30 sec, and 72°C for 7 min; and a final extension of 72°C for 7 min. Products were gel-purified using the QIAquick Gel Extraction Kit (Qiagen Inc., Valencia, CA) and sequenced from both ends with primers CNL12 and CNS1. A primer walking strategy was employed in an attempt to sequence the entire IGS. However, repetitive runs of guanine and cytosine nucleotides made it difficult to sequence through certain regions, resulting in successful sequencing of approximately 330 nucleotides at the 5′-end of IGS and approximately 875 nucleotides at the 3′-end. The 5′- and 3′-ends of the IGS for several fungi that are closely related to *O. ophiodiicola* (Table 1) were also amplified and sequenced as described above. All newly generated sequences for this study (which included sequences from 10 isolates of *O. ophiodiicola* and eight species of closely related fungi) were deposited in GenBank (Table 1).

### Primer and probe design

The ITS and IGS of multiple strains of *O. ophiodiicola*, along with selected near relatives (Table 1), were aligned using MegAlign™ (Lasergene® 10, DNASTAR, Madison, WI). For the ITS alignment, ITS-1 demonstrated the greatest interspecific variability, but this region also contained numerous single nucleotide polymorphisms (SNPs) between strains of *O. ophiodiicola* and appeared to be an unsuitable target for the development of an *O. ophiodiicola*-specific PCR capable of detecting all strains of the fungus. ITS-2 was more conserved among different strains of *O. ophiodiicola* but still demonstrated interspecific divergence. Thus, ITS-2 was targeted for assay development. For the IGS alignment, both the 5′- and 3′-ends of the IGS sequence of *O. ophiodiicola* were highly divergent from those of its nearest neighbors’ (excluding portions of the sequences that represented and flanked the 3′-end of the 28S ribosomal RNA (rRNA) gene and the 5′-end of the 18S rRNA gene; these areas were avoided for assay development). Different strains of *O. ophiodiicola* shared high identity within the IGS, but intraspecific variation was observed at numerous SNPs throughout the regions sequenced. Areas of the IGS that were most conserved among all evaluated strains of *O. ophiodiicola* were targeted for development of an IGS PCR.

**Figure 1:**
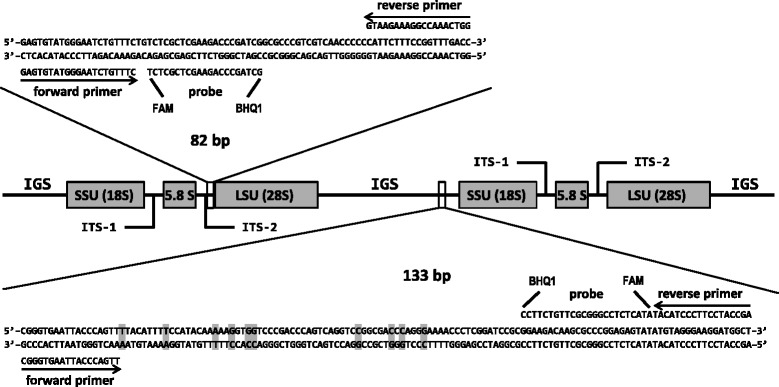
Diagram of fungal ribosomal RNA regions and areas targeted for *Ophidiomyces ophiodiicola*-specific real-time PCR assays. One assay targets the 3′-end of the internal transcribed spacer region 2 (ITS-2) while the second assay targets the 3′-end of the intergenic spacer region (IGS). Expanded sequences (representing the type isolate of *O. ophiodiicola*) show the amplicons produced by each PCR and the primer and probe binding sites. Shaded boxes within the sequences represent nucleotide positions that vary between strains of *O. ophiodiicola*.

Primers and probes were designed using the online PrimerQuest® program (Integrated DNA Technologies, Coralville, IA) [15]. The qPCR option was selected and design parameters were modified such that the program chose primers with a minimum calculated melting temperature (T_m_) of 57°C, an optimum T_m_ of 60°C, and a maximum T_m_ of 65°C; probe T_m_ was set between 64°C and 75°C, with an optimum T_m_ of 70°C. Several primer/probe sets meeting these criteria were identified for both ITS and IGS; however, in each case the binding sites for the set either spanned one to several SNPs among strains of *O. ophiodicola* or resided within an area where the oligos might bind to DNA from non-target species. Thus, binding positions of some oligos were modified slightly from the original PrimerQuest® output such that they would bind more specifically to DNA from *O. ophiodiicola* while also residing within regions conserved among all analyzed strains of the fungus. Potential formation of secondary structure, self-binding, and T_m_ were also considered when designing the oligos. The newly designed primer and probe sequences for the ITS assay were: Oo-rt-ITS-F (forward primer) 5′-GAGTGTATGGGAATCTGTTTC-3′; Oo-rt-ITS-R (reverse primer) 5′-GGTCAAACCGGAAAGAATG-3′; Oo-rt-ITS-P (probe) 5′-(FAM)TCTCGCTCGAAGACCCGATCG(BHQ-1)-3′. The newly designed primer and probe sequences for the IGS assay were: Oo-rt-IGS-F (forward primer) 5′-CGGGTGAATTACCCAGTT-3′; Oo-rt-IGS-R (reverse primer) 5′-AGCCATCCTTCCCTACAT-3′; Oo-rt-IGS-P (probe) 5′-(FAM)ATACTCTCCGGGCGCTTGTCTTCC(BHQ-1)-3′ (Figure 1). All primers and probes were searched against the GenBank database using the Basic Local Alignment Search Tool (BLAST) [16]. No sequences in GenBank (excluding those of *O. ophiodiicola*) were a perfect match to the primers or probes, and those sequences that were most similar to a given oligo did not demonstrate significant similarity with the other two oligos in the set.

### Assay development

Real-time PCR was performed on the Applied Biosystems® 7500 Fast Real-Time PCR System. Master mix (QuantiFast Probe PCR + ROX Vial Kit, Qiagen, Inc, Valencia, CA) was prepared according to the manufacturer’s specifications. Included in the 25 μl reaction volume was 12.5 μl 1X QuantiFast Probe Master Mix, 0.5 μl 1X ROX dye solution, 5 μl template DNA, 0.5 μl of each 20 μM primer stock solution (final reaction concentration 0.4 μM), 0.25 μl 20 μM probe stock solution (final reaction concentration 0.2 μM), and 0.5 μl of 20 μg/μl Bovine Serum Albumin (BSA) solution (Sigma-Aldrich, St. Louis, MO; final reaction concentration of 0.4 μg/μl) to mitigate possible PCR inhibitors. Oligos were synthesized by a commercial company (BioSearch Technologies, Inc., Novata CA). Standard cycling conditions were used for both assays: 95°C for 3 minutes, 95°C for 3 seconds, 60°C for 30 seconds for a total of 40 cycles. To standardize cycle threshold (C_t_) values across PCR runs, the threshold was set at four percent of the maximum background-subtracted fluorescence (ΔRn). At this threshold, sample amplification curves approached maximum efficiency, negative controls were below the threshold, and weak positives exceeded the threshold. Efficiency along individual amplification curves was determined by calculating the slope of five consecutive ΔRn fluorescence readings [17]. Samples that crossed the threshold at ≤ 36 cycles for the ITS assay and ≤ 38 cycles for the IGS assay were considered positive for diagnostic purposes. These threshold cutoffs were selected because standard curve results indicated that the assays performed inconsistently when template DNA concentrations yielded C_t_ values above 36 and 38 for each assay, respectively. For each plate run (including those for assay qualification), a negative control (water added in place of template) and standard curve of gDNA (5 X 10^5^, 5 X 10^4^, 5 X 10^3^, 5 X 10^2^, and 50 fg) isolated from the type isolate of *O. ophiodiicola* (CBS 122913) were included.

The efficiency, precision, and detection limit of the PCR assays were evaluated based on a ten-fold standard curve dilution series of gDNA (5.0 ag to 5.0 ng) isolated from the type isolate of *O. ophiodiicola*. Efficiency was calculated from the slope of the best fit line of C_t_ value plotted against the log-transformed quantity (fg) of DNA template, (efficiency = −1 + 10 ^(−1/slope)^). The standard curve was run in triplicate, and precision was evaluated based on the expectation that the standard deviations not exceed one C_t_ value. The detection limit was the gDNA concentration at which all three replicates reliably amplified.

### Assay qualification

Consistent detection assays should amplify and capture all *O. ophiodiicola* genetic variants with minimal differences in C_t_ values among those variants. To assess consistency of the real-time PCR assays, 5 pg of gDNA from seven isolates (CBS 122913, UAMH 6218, UAMH 6642, UAMH 6688, UAMH 9985, UAMH 10296, UAMH 10768; Table 1) representing all known ITS and IGS sequence variants of *O. ophiodiicola* were tested in duplicate.

To ensure that the assays were specific to *O. ophiodiicola*, the primer and probe sets were tested against a panel of eight closely related fungal species (Table 1). These included several reptile-infecting taxa for which phylogenetic relationships were recently resolved [6], a *Chrysosporium* sp. isolated from a snake (CBS 629.79), and other near neighbors within the Onygenales group. The assays were also screened against representative isolates of 20 fungal species that were commonly isolated from the skin of snakes submitted to the U.S. Geological Survey – National Wildlife Health Center (NWHC) for diagnostic evaluation (Table 2).

The sensitivity of the real-time PCR assays were determined using tissue samples collected from 70 wild snakes with skin lesions from the eastern half of the U.S. Samples originated from submissions to the NWHC that included snakes carcasses found in the wild, snakes that were humanely euthanized, and non-lethally collected skin biopsies. These samples ranged from deep dermal granulomas and full cross-sections of infected skin (collected from dead snakes or as biopsies) to superficial epidermal crusts (collected as scale clippings or from lesions on shed skin). Corresponding sections of tissue from 50 of the 70 snakes had tested positive for *O. ophiodiicola* by culture analyses conducted at the NWHC. Sections of snout, dorsal, and ventral skin from 16 snakes without signs of skin infection and that had tested negative for the presence of *O. ophiodiicola* by culture analyses were analyzed as disease-negative samples. DNA was extracted from tissues approximately 2 mm^3^ in size using the Gentra®Puregene® Tissue Kit, “solid tissues protocol”. For the lysis procedure, 0.5 mg/ml proteinase K was added to the lysis buffer containing tissue, and the mixture was digested for 1.5 to 3 hours. Tissues were then macerated with a pestle and further digested overnight. The RNase treatment step was omitted, but the procedure was otherwise carried out according to the manufacturer’s instructions. A negative control (no tissue added) extraction was performed to ensure that reagents were not contaminated. Extracted DNA was diluted ten-fold and the diluted template used for real-time PCR as described above. To test for inhibition, the snout, dorsal, and ventral skin DNA extracts of four randomly selected PCR- and culture-negative snakes were spiked with 5 pg *O. ophiodiicola* gDNA. The C_t_ values of the spiked samples were then compared to C_t_ values of the 5 pg standard curve replicates on the same plate to ensure that amplification was equivalent. The sensitivity of each PCR was compared to that of the culture-based analyses with McNemar’s Test in SigmaPlot 11.2 (Systat Software, Inc., San Jose, CA).

Amplified DNA from 5 randomly selected PCR- and culture-positive samples and all culture-negative samples that produced positive PCR results were sequenced to confirm that the amplicons matched the targeted region. Briefly, amplicons were cloned using the Invitrogen™ TOPO® TA Cloning® Kit with the pCR™ 2.1-TOPO® vector and TOP10 competent cells (Life Technologies, Carlsbad, CA), the clones screened by PCR with vector-specific primers, and the resulting PCR products sequenced according to the manufacturer’s instructions.

### Ethics statement

All carcass collection, euthanasia, and biopsy sampling procedures were performed under the authority of other agencies in accordance with applicable state laws; specimens were submitted for routine diagnostic analyses that did not require further review by NWHC institutional committees. Collection of scale clippings from live snakes by NWHC personnel was conducted under NWHC Institutional Animal Care and Use Committee protocol EP130117 and was done in compliance with all necessary permissions and permitting requirements. Animals sampled for this study were wild and not client-owned; thus no owner consent was required.

## Results

### Assay efficiency and precision

**Figure 2:**
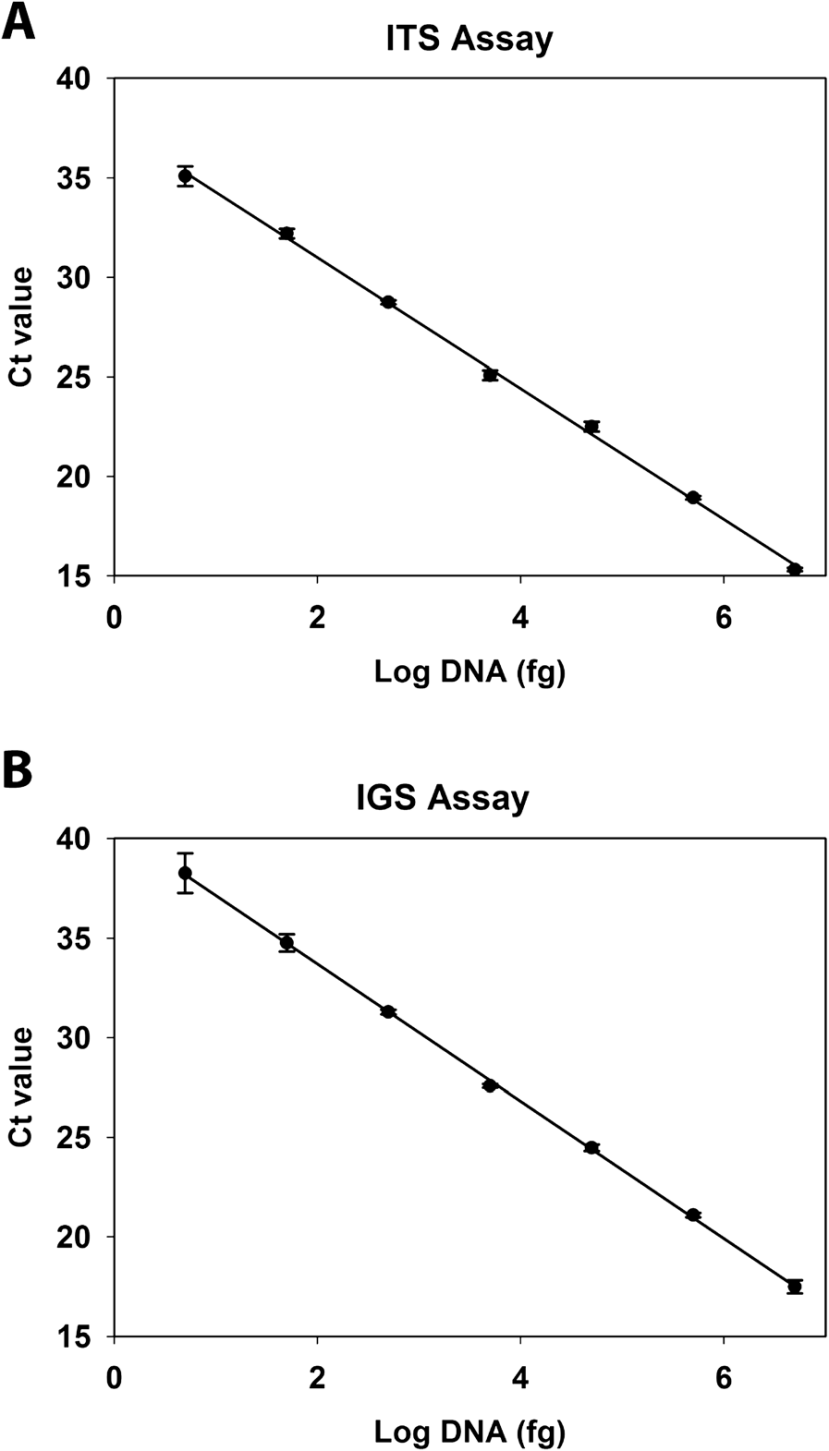
Standard curves for *Ophidiomyces ophiodiicola* real-time PCR assays. Genomic DNA (gDNA) isolated from a pure culture of the type isolate of *O. ophiodiicola* was quantified, serially diluted, and used as template. All samples were run in triplicate with all points depicting mean C_t_ values and error bars representing standard deviation. The assays targeting (**A**) the internal transcribed spacer region (ITS assay) and (**B**) intergenic spacer region (IGS assay) of *O. ophiodiicola* were both linear over seven logs, ranging from 5 fg to 5 ng template gDNA (ITS assay: R^2^ = 0.998; IGS assay: R^2^ = 0.997).

The standard curve efficiency of both the ITS and IGS PCR assays were within the accepted range of 90%-110% [18]. Specifically, the ITS standard curve was 101.41% efficient with an R^2^-value of 0.998. The IGS standard curve had an efficiency of 95.02% and an R^2^-value of 0.997 (Figure 2). All standard curve replicates were precise, varying from one another by less than one C_t_ value (standard deviations for replicates of the ITS assay ranged from 0.08 to 0.49; those of the IGS assay were between 0.09 and 0.44). The detection limit for both assays was 5 fg of purified gDNA. The ITS assay, on average, yielded C_t_ values that were 2.44 cycles lower than that of the IGS assay.

### Assay specificity and sensitivity

The specificities of the ITS and IGS assays were tested using a panel of eight fungi closely related to *O. ophiodiicola* and 20 additional fungi that are commonly associated with snake skin. No discernible amplification was detected when DNA extracted from these fungi were used as template. The assays did, however, amplify target DNA from all known genetic variants of *O. ophiodiicola*. The C_t_ values among these variants were very similar (standard deviations of 0.63 and 0.64 for the ITS and IGS assays, respectively), indicating that performance of the PCR did not vary by strain.

Both assays also performed identically when they were used to screen snake tissue samples for the presence of *O. ophiodiicola*. Forty-nine of the 50 (98%) tissue samples that were culture-positive for *O. ophiodiicola* were also positive by PCR. The remaining sample showed no amplification after 40 cycles with the ITS assay, but had a C_t_ of 39.12 with the IGS assay (above the 38 cycle cut-off). Eight of the 20 tissue samples from snakes that had gross lesions suggestive of SFD but that were culture-negative for *O. ophiodiicola* were PCR-positive. One additional sample had a C_t_ of 38.76 with the IGS assay, but all other samples yielded no amplification. Of the 48 total samples from animals without clinical signs of SFD (3 pieces of skin from each of 16 snakes), a single sample (snout skin from one snake) was PCR-positive by both assays. An additional sample (dorsal body skin) from the same animal, as well as ventral body skin from a second animal, demonstrated amplification with the ITS assay, but had C_t_ values over 36. No amplification was observed in the negative extraction control sample. The inability of the two assays to perform equivalently at high C_t_ values and the questionable clinical significance of extremely low levels of *O. ophiodiicola* DNA further demonstrated the practicality of setting the C_t_ threshold at 36 (ITS assay) and 38 (IGS assay) for diagnostic purposes. When the two detection methods were compared, the PCR assays proved to be more sensitive than culture-based detection methods (χ^2^ = 4.900, p = 0.027).

For all runs, negative controls performed as expected and standard curves were linear. The C_t_ values of the spiked negative extracts were within one C_t_ of the standard curve replicates containing the same amount of gDNA; thus, there was no evidence of inhibition. All PCR products that were sequenced were 100% identical to the desired ITS or IGS amplicons, indicating the positive results were not due to cross-amplification of non-target DNA.

## Discussion

Advances in diagnostics and research related to *O. ophiodiicola* and its associated infections have been hampered by lack of reliable and sensitive techniques to detect the fungus. We designed two real-time PCR methods to rapidly identify the fungus in clinical samples. Both PCR assays proved to be more sensitive than culture-based methods for detecting *O. ophiodiicola*. Specifically, PCR yielded negative results for only 2% of samples that were culture-positive for *O. ophiodiicola*; and detected the fungus in 8 of 20 (40%) culture-negative samples that had been collected from snakes with clinical signs suggestive of SFD. In four of the remaining 12 snakes with grossly visible skin infections that were culture- and PCR-negative, fungi possessing morphological characteristics inconsistent with *O. ophiodiicola* were observed by histopathology; in three additional samples, fungi consistent with *O. ophiodiicola* were seen but could not be definitively identified microscopically; and the five remaining samples consisted of scale clippings and thus were unsuitable for histopathologic examination. Since no “gold standard” method for detection of *O. ophiodiicola* currently exists, it is difficult to ascertain whether other microorganisms caused most of the infections that were PCR-negative. Bacteria, other fungi, and noninfectious processes are known to cause clinical signs that are grossly indistinguishable from those associated with *O. ophiodiicola* [2], and it is likely that at least some PCR-negative samples represented infections caused by other etiological agents. In histopathology, the cutaneous fungal invasion that characterizes SFD is often localized. Thus, it is also possible that dividing tissues for different types of analyses may have resulted in allocation of tissue for PCR that did not capture the active lesion or contained insufficient numbers of fungal elements. This likely explains the 2% false-negative rate for culture-positive samples and highlights the importance of targeting an active area of the lesion for PCR testing. Nonetheless, these data indicate that the PCR assays represent the most sensitive tool currently available to detect *O. ophiodiicola*.

In our PCR screen, one of 16 (~6%) snakes without clinical signs of SFD tested positive for *O. ophiodiicola* (C_t_ values of 29.16 and 30.94 for the ITS and IGS PCR assays, respectively). To rule out contamination as a source of these PCR-positive results, we sampled additional tissues from the same animal (*i.e.*, chin skin, spectacle, and two pieces of snout skin). PCR indicated that *O. ophiodiicola* was also present on the chin skin and spectacle, suggesting that contamination was an unlikely explanation for the positive results. A sample from one additional snake without lesions that was classified as PCR-negative also showed evidence of amplification above the diagnostic threshold cut-off. Sequenced amplicons of the PCR-positive snake without clinical signs of infection and of the additional culture-negative snake with a C_t_ value above the diagnostic cut-off (neither of which were suspected to have had SFD) were a 100% match to *O. ophiodiicola*. Whether such detections represent early stages of colonization/infection by the fungus or indicate the potential of some snakes to harbor low levels of *O. ophiodiicola* in the absence of disease manifestation is unclear, but the latter seems more plausible. Paré et al. [3] reported culturing *O. ophiodiicola* from shed skin of less than 1% of captive snakes without clinical signs of skin infection, and the authors speculated that the fungus occurs only rarely on asymptomatic animals. However, the low detection rate in that study may have been due to the insensitivity of culture techniques, particularly when the fungus is at low abundance. With its much greater sensitivity, PCR has great utility in research aimed at better parsing out the association between *O. ophiodiicola* and disease, prevalence of the fungus on asymptomatic animals, and the importance of pathogen load in disease development.

The PCR assays also provide an important tool to assist with identification of *O. ophiodiicola*-associated infections in the clinical setting. The rapid turnaround time of PCR (less than 24 hours) can provide important preliminary results that allow for immediate treatment of animals suspected to have SFD, can be used to help verify whether fungal hyphae observed in histopathology are *O. ophiodiicola*, and will provide an alternative to morphological- or DNA sequencing-based identification of pure cultures of *O. ophiodiicola*. Although the fungus may occasionally be detected on the skin of snakes that do not have SFD, animals with gross lesions tend to have heavier burdens of *O. ophiodiicola*. For example, based on average C_t_ values, snakes with infections known to be associated with *O. ophiodiicola* (*i.e.*, culture-positive) had approximately 1,000 times more *O. ophiodiicola* DNA detected in their tissues than PCR-positive snakes without clinical signs of SFD. When the active lesion is sampled, C_t_ values can generally be expected to be low if *O. ophiodiicola* is associated with the infection. Furthermore, snakes with severe skin infections typically have multiple lesions on the body that can be non-lethally sampled. By sampling more than one lesion (or several areas of a single large lesion), clinicians can be confident that PCR results represent true-positive or true-negative detections.

While the PCR assays we describe are useful diagnostic tools, it is important to note that histopathology in conjunction with PCR is necessary to make definitive diagnoses that accurately distinguish disease from mere presence of the pathogen. However, there are applications in which simply confirming the presence of *O. ophiodiicola* (irrespective of actual disease) may be useful. For example, PCR would provide a much more sensitive method to screen for asymptomatic carriers of the fungus during the importation and quarantine process at zoological parks and reptile collections as well as to screen environmental samples for potential contamination with *O. ophiodiicola*. For such applications, laboratories may wish to set the PCR threshold at 40 or more cycles to detect very low levels of the fungus. In addition, multiple samples should be tested to reduce the likelihood of false-negative results.

Both PCR assays described in this report target portions of the rRNA gene complex. This gene region occurs as multiple copies within the fungal genome, affording high sensitivity to assays that target the rRNA gene complex. For example, Muller et al. [19] estimated that the lower limit of detection of a real-time PCR assay specific to the IGS of *Pseudogymnoascus destructans* (the causative agent of bat white-nose syndrome) was approximately 0.1 genome equivalents. At this time, neither the number of copies of rRNA genes on, nor the size of, the *O. ophiodiicola* genome is known, making it difficult to predict the lower limit of detection in genome equivalents. However, detection limits based on purified gDNA were similar between our assays and that of Muller et al. [19]. Different strains of *O. ophiodiicola* apparently have a similar number of rRNA gene region copies as C_t_ values were consistent between them when known amounts of gDNA were used as template. This indicates that the PCR assays have great potential for research that requires quantification of the fungus in samples. However, more work is necessary to more definitively determine how C_t_ values translate to cell-equivalents.

The overall performance of the ITS and IGS assays were similar, but each assay is optimally suited for different applications. The ITS assay is the more robust option for diagnostic work, particularly for laboratories that may be receiving samples from captive snakes. Although both assays performed well against all currently known strains of *O. ophiodiicola*, there are undoubtedly more strains yet to be characterized. The more highly conserved nature of ITS between strains, however, makes the ITS assay more likely to detect novel and potentially divergent genetic variants of *O. ophiodiicola*. Such variants are more likely to be encountered in captive snakes or wild snakes from outside eastern North America (since most characterized strains upon which the assays were designed originated in the eastern U.S.). The IGS exhibits greater DNA sequence variation between strains, increasing the possibility that novel genetic variants may have SNPs in oligo binding sites within the IGS. Thus while there is no evidence to indicate that the IGS assay is unsuitable for diagnostic work, the use of the ITS assay for this purpose represents the more conservative approach.

For screening environmental samples for *O. ophiodiicola*, the IGS assay is recommended. While both assays discriminated between *O. ophiodiicola* and its closest known relatives, we cannot rule out the possibility that there are other uncharacterized environmental fungi that are genetically similar to *O. ophiodiicola* and that could cross-react with the ITS assay primers and probe. For example, CANV complex species within polytypic genera (*e.g.*, *Nannizziopsis* and *Paranannizziopsis*) may share over 97% DNA sequence identity in the ITS [6]; if such small differences in ITS sequences existed between *O. ophiodiicola* and other hypothetical species of *Ophidiomyces*, the ITS assay would be unlikely to differentiate them. While sequencing of amplicons in this study indicated no evidence that such closely-related fungi exist on the skin of snakes, environmental samples may harbor much more diverse fungal communities. For example, the original ITS-based PCR used to detect *P. destructans* performed well when testing bat tissues, but was unsuitable for discriminating the pathogen from a wide variety of closely-related, nonpathogenic, and previously undiscovered *Pseudogymnoascus* spp. in soil samples [20,21]; this subsequently prompted the development of a more specific PCR assay targeting the IGS of *P. destructans* which exhibits much higher interspecific variation than the ITS [19]. Such high variation in the region makes it highly unlikely that the IGS assay described herein would cross-amplify DNA from fungi other than *O. ophiodiicola*, including very closely related hypothetical taxa that are yet to be discovered. The IGS assay also has utility for research that requires differentiation among strains of *O. ophiodiicola* – because the amplicon produced by the IGS PCR encompasses numerous SNPs (Figure 1), sequencing the amplicon can distinguish between some genetic variants of the fungus.

## Conclusions

The increase of CANV-associated infections in captive reptiles and the emergence of SFD in wild snakes have elicited concern over the future health of both captive and wild snake populations. As clinicians attempt to diagnose an increasingly common disease and researchers attempt to better understand the disease system, enhanced methods to detect the fungus linked to these infections will be essential. The PCR assays described herein provide important tools to advance SFD diagnostics, support surveillance efforts, and aid researchers trying to better understand the biology of *O. ophiodiicola*, the intricacies of host-pathogen dynamics, and the drivers of SFD emergence.

## Abbreviations

BHQ-1: Black Hole Quencher®-1
BLAST: basic local alignment search tool
BSA: bovine serum albumin
CANV: *Chrysosporium* anamorph of *Nannizziopsis vriesii*
CBS: Centraalbureau voor Schimmelcultures
C_t_: cycle threshold
DNA: deoxyribonucleic acid
ΔRn: background-subtracted fluorescence
FAM: 6-carboxyfluorescein
gDNA: genomic deoxyribonucleic acid
IGS: intergenic spacer region
ITS: internal transcribed spacer region
NWHC: U.S. Geological Survey - National Wildlife Health Center
PCR: polymerase chain reaction
RNA: ribonucleic acid
rRNA: ribosomal ribonucleic acid
ROX: 6-carboxyl-x-rhodamine
SFD: snake fungal disease
SNPs: single nucleotide polymorphisms
T_m_: melting temperature
UAMH: University of Alberta Microfungus Collection and Herbarium
